# A Study of Major and Minor Complications of 1500 Transvenous Lead Extraction Procedures Performed with Optimal Safety at Two High-Volume Referral Centers

**DOI:** 10.3390/ijerph181910416

**Published:** 2021-10-03

**Authors:** Łukasz Tułecki, Anna Polewczyk, Wojciech Jacheć, Dorota Nowosielecka, Konrad Tomków, Paweł Stefańczyk, Jarosław Kosior, Krzysztof Duda, Maciej Polewczyk, Andrzej Kutarski

**Affiliations:** 1 Department of Cardiac Surgery, The Pope John Paul II Province Hospital of Zamość, 22-400 Zamość, Poland; luke27@poczta.onet.pl (Ł.T.); dornowos@wp.pl (D.N.); konradtomkow@wp.pl (K.T.); paolost@interia.pl (P.S.); 2 Department of Physiology Pathophysiology and Clinical Immunology, Collegium Medicum, The Jan Kochanowski University, 25-317 Kielce, Poland; 3 Department of Cardiac Surgery, Świętokrzyskie Cardiology Center, 25-736 Kielce, Poland; 4 2nd Department of Cardiology, Silesian Medical University, 41-808 Zabrze, Poland; wjachec@interia.pl; 5 Department of Cardiology, Masovian Specialist Hospital of Radom, 26-617 Radom, Poland; jaroslaw.kosior@icloud.com (J.K.); kadeder@gmail.com (K.D.); 6 Department of Microbiology, Collegium Medicum, Jan Kochanowski Univeristy, 25-369 Kielce, Poland; Maciek.polewczyk@gmail.com; 7 Intensive Care Unit, Świętokrzyskie Cardiology Center, 25-736 Kielce, Poland; 8 Department of Cardiology, Medical University, 20-059 Lublin, Poland; a_kutarski@yahoo.com

**Keywords:** transvenous lead extraction, minor and major complications, cardiac laceration/vascular tear, epicardial fluid, tricuspid valve damage

## Abstract

*Background:* Transvenous lead extraction (TLE) is the preferred management strategy for complications related to cardiac implantable electronic devices. TLE sometimes can cause serious complications. *Methods:* Outcomes of TLE procedures using non-powered mechanical sheaths were analyzed in 1500 patients (mean age 68.11 years; 39.86% females) admitted to two high-volume centers. *Results:* Complete procedural success was achieved in 96.13% of patients; clinical success in 98.93%, no periprocedural death occurred. Mean lead dwell time in the study population was 112.1 months. Minor complications developed in 115 (7.65%), major complications in 33 (2.20%) patients. The most frequent minor complications were tricuspid valve damage (TVD) (3.20%) and pericardial effusion that did not necessitate immediate intervention (1.33%). The most common major complication was cardiac laceration/vascular tear (1.40%) followed by an increase in TVD by two or three grades to grade 4 (0.80%). *Conclusions:* Despite the long implant duration (112.1 months) satisfying results without procedure-related death can be obtained using mechanical tools. Lead remnants or severe tricuspid regurgitation was the principal cause of lack of clinical and procedural success. Worsening TR(Tricuspid regurgitation) (due to its long-term consequences), but not cardiac/vascular wall damage; is still the biggest TLE-related problem; when non-powered mechanical sheaths are used as first-line tools.

## 1. Introduction

Transvenous lead extraction (TLE) is considered an integral part of the management strategy for complications related to the presence of cardiac implantable electronic devices (CIED) [1,2,3,4,5].Due to the foreign body reaction and extensive fibrotic scarring around the leads [6,7], TLE can sometimes cause severe damage to the veins and heart as manifested by bleeding into the mediastinum or right pleural cavity, or acute pericardial effusion depending on the location of the tear [1,2,3,4,5,8,9,10,11,12].Another problem we face in TLE is the real risk of tricuspid valve damage (TVD) with worsening tricuspid regurgitation (TR). The problem of TVD was omitted in the older guidelines [1,2,3,4] and addressed only just in the recent ones [4,5]. There are several reports concerning the role of cardiac surgery and cardiac anesthesiology in the management TLE-related cardiovascular injuries [8,9,10,11,12] and TLE effectiveness [13,14,15,16,17,18], but there is no comprehensive investigation of cardiac laceration/vascular walltear (CVWT) and TVD as major and minor complications of lead extraction. Some investigators reported TR increase without any reference to minor and major TLE complication [19,20,21,22,23,24,25]. At our center all cases of symptomatic cardiac tamponade were managed with sternotomy, therefore we were able to provide more precise information about the location of tears. Additionally, continuous TEE (Trans esophageal echocardiography) monitoring enabled a more rapid and accurate assessment of worsening TR.

The aim of the present study was to determine the occurrence and describe in detail cardiac/vascular wall rupture and TV damage as a form of major and minor complications related to TLE. Particular attention was paid to the worsening of tricuspid regurgitation and the difficulty in classification.

## 2. Material and Methods

### 2.1. Study Population

This study was a post hoc analysis of the clinical data of 1500 patients undergoing transvenous lead extraction at two high-volume TLE centers between June 2015 and April 2021. All extraction procedures were performed by the same first operator and nurses in compliance with the same optimal safety regulations. All information relating to patients and procedures was entered into a computer on an ongoing basis. Conventional mechanical sheaths were the first-line tools; powered rotational mechanical sheaths and other instruments were used as the second option. Laser sheaths were not used at our centers.

### 2.2. Lead Extraction Procedure

Lead extraction procedures were defined according to the most recent guidelines on the management of lead-related complications (HRS 2017 and EHRA 2018) [4,5]. Indications for TLE and type of periprocedural complications were defined according to the 2017 HRS Expert Consensus Statement on Cardiovascular Implantable Electronic Device Lead Management and Extraction [4].

Most removal procedures were performed using non-powered mechanical systems such as polypropylene Byrd dilator sheaths (Cook^®^ Medical, Leechburg, PA, USA), if only possible via the implant vein. If technical difficulties arose, additional tools such as Evolution (Cook^®^ Medical, Leechburg, PA, USA), TightRail (Spectranetix, Colorado Springs, CO, USA), lassos, basket catheters and/or alternative venous approaches were utilized. The excimer laser was not applied.

All TLE procedures were performed following the same organizational model. The operating team consisted of a very experienced TLE operator, cardiac surgeon, anesthesiologist and echocardiographist. The procedures were performed in a hybrid room or an operating room on the cardiac surgery ward, with a full range of equipment for an emergency rescue.

The SAFeTY TLE score was used to assess the risk for the occurrence of major complications related to TLE [26] using an online calculator, available at http://alamay2.linuxpl.info/kalkulator/ (accessed on 11 September 2021).

### 2.3. TEE Monitoring during TLE

Echocardiography, especially continuous transesophageal echocardiography (TEE) is a very useful tool that improves the safety of TLE procedures [27]. In this study, TEE was performed by two experienced echocardiographers using Philips iE33, GE Vivid S 70 and GE Vivid E-95(GE Vivid S 70 and GE Vivid E-95—both GE Medical Systems, San Francisco, CA, USA) machines equipped with X7-2t Live 3D or 6VT-D probes. All examinations were archived and information was stored in a computer database. The applications of TEE included preprocedural assessment of lead position with particular emphasis on the presence of additional masses on the leads, evaluation of tricuspid valve function and navigation of lead removal, whereas postoperative TEE was used to determine procedure effectiveness and possible complications [28,29,30].

Intraoperative TEE allowed visualization of direct pulling on the heart during lead extraction, helping explain a frequent drop in blood pressure due to right ventricular collapse [27,28,29]. A very important role of TEE is to rapidly detect accumulation of blood in the pericardial sac. If the walls of the heart are damaged, TEE can help locate the perforation site by identifying the segment of the wall on which the greatest pulling force is exerted. Additionally, TEE provides information not only on the volume of blood in the pericardial sac and the diastolic function of the right ventricle, but also on the location of fluid and blood clots in terms of the chances of successful pericardial puncture [28,29]. The postoperative phase of TLE includes the evaluation of tricuspid valve function and the assessment of lead remnants, residual vegetations and free-floating fragments of fibrous encapsulation.

### 2.4. Management of Symptomatic Cardiac Laceration/Vascular Wall Tear

From the very beginning all TLE procedures in the two hospitals have been performed in compliance with the available recommendations [2,3,4,5] in terms of the venue, participation of cardiac surgeons and anesthesia teams, continuous blood pressure monitoring, TEE monitoring and measurements of partial pressure of carbon dioxide in the expired air. The availability of cardiac surgeons with surgical instruments and nursing staff made an attempt at pericardiocentesis unreasonable, therefore the preferred choice was proper surgery without further delay (except one patient with borderline hemodynamic values). With this strategy, all urgent interventions were successful and effective, none of the patients died. Only in 2 out of 21 patients with cardiac laceration/vascular wall tear (CVWT) in our series required the use of cardiopulmonary bypass pump (CPB) to support the circulation. One patient required simultaneous urgent tricuspid valve repair and another one reconstruction of the superior vena cava together with TV repair and replacement. In the remaining 19 patients CPB was not necessary.

### 2.5. Assessment of Tricuspid Valve Damage

The postprocedural phase of TEE monitoring includes reassessment of cardiac/vascular wall injuries and as exact as possible reevaluation of TV function (including the comparison with baseline findings). The mid-esophageal, inferior esophageal and modified transgastric views were applied to visualize the right heart chambers and the tricuspid valve [30]. For visualization of the entire cardiac anatomy and assessment of the course of the lead non-standard imaging planes were sometimes required. The projections and consecutive stages of echocardiographic monitoring were described in detail in previous publications [27,28,29,30].

### 2.6. Presentation of Study Results

Categorical variables were presented as numbers and percentages, and continuous variables were expressed as the mean and standard deviation (SD)

#### Approval of the Bioethics Committee

The study was carried out in accordance with the ethical standards of the 1964 Declaration of Helsinki. All patients gave their informed written consent to undergo TLE and to use anonymous data from their medical records, which was approved by the Bioethics Committee at the Regional Chamber of Physicians in Lublin no. 288/2018/KB/VII.

## 3. Results

The study population consisted of 1500 patients (mean age 68.11 years, 39.86% of women). The mean dwell time of the oldest extracted lead per patient was 112.1 months, the sum of lead dwell times was 17.01 years. The total number of major and minor complications was 33 (2.20%) and 115 (7.67%), respectively. Complete procedural success was obtained in 96.13%, partial radiographic success in 3.07%, whereas clinical success in 98.93% of the 1500 patients/procedures (Table 1).

The most important reason for the absence of clinical and procedural success was the lack of complete procedural success (a non-extractable tip of the lead or lead fragments < 4 cm left behind in the heart). However, even the presence of lead remnants can be regarded as clinically acceptable, if procedure indications are non-infectious. In infectious cases incomplete lead removal is considered as failure to achieve clinical success despite the absence of negative effects on the course of infection. The second reason for no clinical and procedural success was a severe increase in tricuspid regurgitation meeting the echocardiographic criteria for cardiac surgery. Severe deterioration of TV function not necessitating surgery was categorized as minor complications. Despite the need to perform 21 surgical rescue interventions there was no procedure-related death that otherwise would be the reason for no clinical or procedural success. It should be emphasized that contrary to popular opinion, the leading TLE-related problem is still worsening TR (due to its long-term consequences) but not cardiac/vascular wall damage when non-powered mechanical sheaths are used as the first-line option (Table 2).

Minor complications after TLE were observed in 115 (7.67%) patients. The most frequent complication was tricuspid valve damage (worsening by two or three degrees but not to grade 4) detected in 43 patients (2.91%). Worsening by one degree only was not considered minor complication because such a difference may be very subtle leading to an error caused for instance by fluid oversupply. Another minor complication in the present study was pericardial effusion not requiring pericardiocentesis or surgical intervention. It was detected in 24 patients (1.60%). Hemodynamic monitoring (TEE, arterial line, breath gas analysis) helped avoid pericardiocentesis despite a transient drop in blood pressure. The third minor complication (1.33%) was blood transfusion related to blood loss during surgery (the need for transfusion of more than one unit of red blood cells). Hematoma at the surgical site requiring drainage (13 patients, 0.87%) and pneumothorax requiring a chest tube (3 patients, 0.20%) or not (1 patient, 0.067%) were less common (Table 3). This study reveals that worsening TR (12 categorized as major complication, 43 as minor complication and 106 not classified as minor complication) remains the biggest challenge in lead extraction technology (Table 3).

Major complications were observed in 33 (2.20%) patients. The rate appears slightly higher than reported by other investigators but one should bear in mind the prolonged lead dwell time per patient (112.1 months) and the sum of lead dwell times (17.01 years) in the present study. The most common major complication in the 33 patients was cardiac laceration/vascular wall tear (22 lesions in 21 patients, 1.40%) followed by severe tricuspid valve damage (by 2 or 3 degrees to grade 4 in 12 patients, 0.80%). Table 4 summarizes the types of cardiac laceration/vascular wall tear (CVWT) in the 21 patients. Right atrial appendage (RAA) rupture (one double) occurred in 8 patients (0.53%), tear of the connection of the right atrium (RA) to the superior vena cava (SVC) in 5 (0.33%) and SVC laceration in 3 patients (0.20%) (in 2 patients caused by a guidewire or a new lead that passed into the right pleura after lead removal). Other injuries were sporadic and included lateral wall tear (double), RA tear and injury to the coronary sinus (CS) ostium and tear of the connection of the RA to the inferior vena cava (IVC), tear of the right ventricular (RV) wall. Summing up, the connection of the SVC to the RA, RAA wall, and the SVC alone was the most common location of CVWT (16 cases out of 21 requiring surgical repair = 76.19%) but the ventricular wall was affected only in 4.8% of all CVWTs (Table 4).

Table 5 provides a succinct description of the TEE findings that preceded the buildup of pericardial fluid, i.e., blood pooling around the heart, changes in arterial blood pressure and CO^2^ in exhaled air since the onset of CVWT to the cessation of bleeding.

The amount of pericardial fluid depends mainly on the size of the rupture but the rapidity with which the bleeding may be stopped depends on the location of the tear. The present study shows that an injury to structures other than the RA (CS, SVC, connection of RA to IVC) was associated with a higher drop in arterial blood pressure and CO^2^ in exhaled air and a much higher blood loss (blood volume drained during rescue operation) than in cardiac tamponade caused by RA damage. All these findings confirm the differences in the clinical manifestations between damage to the RA and injury to other structures, which seem to be more serious and difficult to manage (Table 5).

The less frequently addressed problem is TLE-related TV damage. Worsening TR is a common term for a wide spectrum of echocardiographic images [19,20,21,22,23,24,25]. This procedure-related TV damage is often superimposed on the previously existing TV dysfunction and it may be difficult to discern the new from the old. The role of an echocardiographer is relatively simple: to describe as exactly as possible TV function and changes from baseline. However, physicians generating inputs to medical databases and preparing discharge summaries may face the problem of how to categorize the worsening of TR: as a non-significant phenomenon (the borderline diagnosis)? minor complication? or major complication? All major complications have to be discussed with the cardiac surgeon regarding the TV replacement.

In the present study, mild TR (grade 1) wasa very frequent finding (49.43% of patients) prior to TLE. Moderate (grade 2), intermediate (grade 3) and severe (grade 4) forms of TR were less common (21.75%, 17.24% and 7.68%, respectively). Changes in the severity of regurgitation after TLE included both an increase (in 10.09% of patients) and a decrease in TR (in 10.30% of patients). The most common finding about TV function after TLE was either worsening or improvement by one degree. It may indicate that the differences by one degree (both directions) are very subtle and are not only “examiner-dependent” but also condition-dependent (rapid fluid intake). On the other hand worsening from grade 3 to grade 4 may have clinical consequences. Lead-dependent TV dysfunction (LDTD) was not the subject of this paper so we did not tackle the issue. Rupture of the chordae tendineae was an additional finding which was observed in moderate and severe TV worsening (43 patients, 2.91%). A moderate increase in TR by 2 or 3 degrees, but not to grade 4 (31 patients 2.10%) was considered minor complication and a significant increase in TR by 2 degrees to grade 4 was considered major complication (Table 6).

In 12 patients worsening TR was classified as major complication. TV replacement (2 acute, 3 late) was performed in 5 patients (41.67% out of 12 i.e., 0.33% out of 1500). Three patients were not classified (slight improvement in control TTE examination) and remained under observation (25.00% out of 12, 0.20% out of 1500). The same number of patients refused TV replacement and they also remained under observation (Table 6).

Table 7 summarizes the occurrence of worsening TR as no complication, minor or major complication after TLE. The data suggests that the worsening of TR by one degree to grade 1 and 2 may be disregarded as a complication. The worsening of TR by one degree from grade 2 to 3 and from grade 3 to 4 remains controversial. Such worsening may be symptomatic.

## 4. Discussion

Transvenous lead extraction is an integral part of the management of CIED-related problems [1,2,3,4,5]. Cardiac and venous injuries during lead extraction are complications with potentially serious consequences. So far there has been no in-depth analysis that would go beyond injury to the SVC/other vessels and attempt to identify TLE-related TV damage (TVD) as minor and major complications of lead extraction.

Despite the long implant duration major complications occurred in 33 out of 1500 (2.20%) patients, whereas minor complications in 115 (7.67%) patients. Complete procedural success was obtained in 96.13%, partial radiographic success in 3.07%, clinical success in 98.93% of 1500 patients/procedures. The most important reason for the absence of clinical and procedural success was the lack of complete radiographic success. The second reason was severe worsening of tricuspid regurgitation meeting echocardiographic criteria for cardiac surgery. Marked deterioration in TV function but not requiring surgery was classified as minor complication. Despite the need for rescue surgery in 21 cases there was no procedure-related death that otherwise would account for the lack of clinical or procedural success. Tricuspid valve damage was the most common minor complication (3.07%). The second minor complication was pericardial effusion not requiring pericardiocentesis or surgical intervention (1.60%). Less frequent minor complications included blood transfusion related to blood loss during surgery (1.33%), hematoma at the surgical site requiring drainage (0.87%) and pneumothorax requiring a chest tube (0.20%) or not (0.067%). Major complications occurred in 2.20% of cases but one should bear in mind that the dwell time of the oldest lead per patient was 112.1 months. The most common major complication was cardiac laceration/vascular wall tear (1.47%) followed by severe tricuspid valve damage (0.80%). The most frequent location of the tear was RAA (0.53%), connection of RA to SVC (0.33%) and SVC (0.20%). Other locations were rare (lateral RA, CS ostium, connection of RA to IVC). There was only one rupture of RVA wall. An injury to structures other than RA (CS, SVC, connection of RA to IVC) was associated with higher drops in arterial blood pressure and CO^2^ in exhaled air and much higher blood losses (blood volume drained during rescue operation) than in tamponade caused by RA damage. All these findings confirm the differences in the clinical manifestations between damage to the RA and injury to other structures, which seem to be more serious and difficult to manage. The worsening of TR related to TLE is a common term for a wide spectrum of echocardiographic images. TR after TLE can either worsen (10.09%) or improve (10.30%). The most frequent finding was worsening or improvement by one degree. Rupture of chordae tendineae was detected as an additional finding in moderate and severe TR worsening (3.21%). Moderate increases in TR by 2 degrees, but not to grade 4 (2.37%) were considered minor complications and significant (by 2 degrees) increase in TR but to grade 4 was considered major complication. In 12 patients worsening TR was classified as major complication. TV replacement was performed (2 acute, 3 late) in 5 patients (41.67% among 12), 3 pts were not selected for surgery (slight improvement in control TTE examinations) and remained under observation. The same number of patients refused TV replacement and they also remained under observation. In terms of classification as lack, minor or major complication after TLE this study suggests that the worsening of TR after TLE by one degree from grade 1 and 2 may be disregarded as a complication. The worsening of TR by one degree from grade 2 to 3 and from grade 3 to 4 remains controversial. Once again, contrary to popular opinion, the leading TLE-related problem is still worsening TR (due to its long-term consequences) but not cardiac/vascular wall damage when non-powered mechanical sheaths are used as the first-line tools.

If excimer laser energy is not applied, major complications other than tear of the SVC and anonymous vein seem to be more frequent [18]. The available guidelines and medical literature describe all forms of cardiovascular wall tear but not worsening TR after TLE [1,2,3,4,5,16,17,18].

There are two large reports concerning vascular and cardiac wall damage during lead extraction using laser technique. Brunner et al. found out that the rate of complications requiring rescue intervention was 0.8% (mean implant duration time 4.9 years in overall cohort). SVC laceration was most frequent (80%), whereas RA and RV wall damage was rare. Hospital mortality was 36% in the group of patients undergoing rescue intervention. Only 44% of patients survived in a good condition and were discharged home [8]. Bashir et al. reported cardiac or venous injuries in 3% of TLE patients, but mean implant duration time was much longer than in the previous study, i.e., 10.8 years. Overall, cardiac tamponade as a devastating injury was detected in 84.8% of cases but no one of the surgeons used pericardiocentesis as a therapeutic modality and urgent sternotomy was performed. Mortality rate in this report was 12.1% [10].

The organization of our TLE teamwork in compliance with maximum patient safety regulations may explain the absence of procedure-related deaths among 1500 patients in spite of the very long mean implant duration time and a relatively frequent need for rescue surgery. Mandatory continuous TEE monitoring during all TLE procedures and measurements of vital signs such as direct arterial blood pressure and capnography allowed us to recognize a serious complication very early, gaining time for proper rescue intervention. The excellent TLE organizational model solves the problem of complication-related deaths but has no or only a small influence on the development of major complications.

Damage to the tricuspid valve during extraction is estimated to range from 3.5% to 15%, and even to 19% [4,5,19,20,21,22,23,24,25]. In this study a clinically insignificant valve dysfunction was detected in 7.18% of cases, whereas significant TV damage that caused worsening TR by 2 or 3 degrees as compared to baseline (before TLE) occurred in 2.91% of patients, which is less than previously reported [4,5,19,20,21,22,23,24,25]. The need for surgical intervention in such cases is rare [19,20,21,22,23,24,25,31].

This study and available evidence [19,20,21,22,23,24,25,31] show that one of the most important TLE safety challenges is still the unsolved problem of TLE-related TV damage which is caused by fibrous adhesion of the lead to the TV leaflet. Excessive pulling on the lead may cause leaflet disruption, but also wrapping of the leaflet around the dilating sheath during rotational lead extraction. Excellent teamwork combined with TEE monitoring may help warn the extractor about potentially harmful situations leading to TV damage [27,28,29,30]. One should also bear in mind that the lead to be removed can be fused to the chordae tendineae or even to the head of the papillary muscle and damages to these structures may go unnoticed.

Continuous TEE monitoring during TLE facilitates the imaging of lead adhesion to the walls of the superior vena cava, tricuspid valve and walls of the right atrium and right ventricle, as well as assessing lead-to-lead adhesion [27]. In this aspect, real time transesophageal echocardiography for the guidance of transvenous lead extraction informs the operator about the danger of manipulations close to delicate cardiac structures and whether immediate modification to the plan of lead removal is necessary in order to prevent the occurrence of unwanted events [28,29]. In turn, postoperative TEE provides information about the results of TLE and helps establish further management [30].

## 5. Study Limitations

There are some limitations of this study. It is the experience of two high-volume centers and the same first operator. The database was prospectively integrated, but analysis was performed retrospectively. The TLE organizational model has not changed since 2015 and takes into account a comprehensive list of safety precautions (hybrid room, cardiac surgeon as co-operator, TEE monitoring, general anesthesia, arterial line etc.). All procedures were performed using all types of mechanical sheaths, but not laser powered sheaths. On the basis of our previous experience (2006–2014) we abstained from pericardiocentesis as a rule to avoid additional risk of complications and delay in open heart surgery as the most effective option.

## 6. Conclusions

Despite the long implant duration time (112.1 months) satisfying outcomes of lead extraction without procedure-related deaths (clinical success 98.93%, procedural success 96.13%) can be achieved using mechanical tools on condition that optimal safety precautions (immediate diagnosis of the event and rescue surgery) are taken into account. The absence of clinical and procedural success is caused by lead remnants or severe worsening of tricuspid regurgitation.

Major complications of TLE are unavoidable and may develop even in 2.20% of cases, minor complications are more frequent (7.67%). Major complications include cardiac laceration/vascular wall tear (1.40%) and severe tricuspid valve damage (0.80%).

The most frequent location of cardiac laceration/vascular wall tear is RAA(Right atrial appendage) (0.35%), connection of RAto VCS (0.33%) and VCS (0.20%). Other tear locations are rare. RAA, connection of RAto VCS and VCS are most frequent locations of CVWT requiring surgical repair (76.19% of cardiovascular wall injury).

Injury to structures other than RA (CS, SVC, connection of RA to IVC) was associated with higher drops in arterial blood pressure and CO^2^ in exhaled air and much higher blood losses.

The most frequent minor complications were tricuspid valve damage (3.07%), pericardial effusion not requiring pericardiocentesis or surgical intervention (1.60%). Less frequent was blood transfusion related to blood loss during surgery, hematoma at the surgical site requiring drainage and pneumothorax.

Worsening of TR after TLE by one degree is a frequent finding (7.18%), worsening by 2 degrees (2.37%) and 3 degrees (0.542%) is rare, but the latter two may require surgery (leaflet repair with TV replacement) or strict follow-up.

The main TLE-related problem is still worsening TR (due to its long-term consequences) but not cardiac/vascular wall damage when non-powered mechanical sheaths are used as the first-line tools.

## Figures and Tables

**Table 1:** Clinical characteristics of the study population.

Characteristics of the Study Population	Count/Average	%/SD
Patient age at TLE [years]	68.11	14.02
Patient age at first system implantation [years]	58.81	15.77
Sex (% of female patients)	598	39.86%
Etiology: IHD, MI	979	65.27%
Underlying disease: cardiomyopathy, valvular heart disease	277	18.47%
Underlying disease: congenital, channelopathies, neurocardiogenic indications, cardiac surgery	243	16.20%
LVEF average [%]	49.26	15.92
Renal failure: patients with creatinine concentration > 2.00/dL	375	25.00%
Previous sternotomy	214	14.27%
Charlson comorbidity index [points]	5.10	3.76
Systemic infection (with or without pocket infection)	230	15.33%
Local (pocket) infection	90	6.00%
Lead failure (replacement)	865	58.33%
Change of pacing mode/upgrading, downgrading	110	7.33%
Other: Abandoned lead/prevention of abandonment, (AF, redundant leads), threatening/potentially threatening lead (loops, free ending, left heart, LDTD) Other (MRI indications, cancer, painful pocket, loss of indication for pacing/ICD) regainingvenous access (symptomatic occlusion, SVC syndrome, lead replacement/upgrading)	193	12.87%
System: pacemaker (any)	1008	67.08%
System: ICD (VVI, DDD)	359	23.97%
System: CRT-D	133	8.87%
Dwell time of the oldest lead per patient [months]	112.1	78.16
Sum of lead dwell times [years]	17.01	13.75
Major complications	33	2.20%
Minor complications	115	7.66%
Complete procedural success	1442	96.13%
Partial radiographic success	46	3.07%
Clinical success	1484	98.93%

Abbreviations: CRT-D—cardiac resynchronization therapy defibrillator, DDD—dual-chamber antibradycardia pacing, ICD—implantable cardioverter defibrillator, IHD—ischemic heart disease, LDTD—lead-dependent tricuspid dysfunction, LVEF—left ventricular ejection fraction, MI—myocardial infarction, SVC—superior vena cava, TLE—transvenous lead extraction, VVI—ventricular demand pacing.

**Table 2:** Effectiveness and safety of TLE.

**Causes of Clinical Failure**	**Patients**	**%**
Clinical success	1484	98.93%
Lead tip left behind– infection	4	0.27%
Significant TLE-related TV damage	12	0.80%
Procedure-related death (intra- or postprocedural)	0	0.00%
All patients	1500	100.0%
**Causes of procedural failure**		
Procedural success	1442	96.13%
Lead tip left behind	16	1.07%
Lead remnant (<4.0 cm)	30	2.00%
Significant TLE-related TV damage	12	0.80%
Procedure-related death (intra-, postprocedural)	0	0.00%
All patients	1500	100.0%

Abbreviations: TLE—transvenous lead extraction, TV—tricuspid valve.

**Table 3:** Analysis of minor complications.

Minor Complications	Patients	%
Number of minor complications	115	7.67%
Tricuspid valve damage by 2 degrees but not to grade 4	43	2.91%
Pericardial effusion not requiring pericardiocentesis or surgical intervention	20	1.33%
Blood transfusion related to blood loss during surgery	17	1.13%
Hematoma at the surgical site requiring drainage	13	0.87%
Arm swelling or lead-induced venous thrombosis resulting in medical intervention	4	0.29%
Pneumothorax requiring a chest tube	3	0.20%
Blood transfusion related to blood loss during surgery	1	0.067%
Arm swelling or lead-induced venous thrombosis	1	0.067%
Tricuspid valve damage + pericardial effusion not requiring pericardiocentesis	1	0.067%
Tricuspid valve damage + hemothorax not requiring a chest tube	1	0.067%
Pericardial effusion + pneumothorax not requiring intervention	1	0.067%
Pericardial effusion not requiring pericardiocentesis + blood transfusion	1	0.067%
Pericardial effusion not requiring pericardiocentesis + vascular repair at lead venous entry site + blood transfusion	1	0.067%
Migrated lead fragment without sequelae	1	0.067%
Femoral vein thrombosis	1	0.067%
Other mixed	5	0.34%
All patients	1500	100.000%

**Table 4:** Analysis of major complications.

**Major Complications**	**Patients**	**%**
Number of major complications	33	2.20%
Hemopericardium:rescue cardiac surgery	17	1.13%
Hemopericardium drainage (pericardiocentesis)	1	0.067%
Hemothorax:rescue cardiac surgery	2	0.13%
Acute heart failure (decrease in BP and contractility as a reaction to guide wire in mediastinum)	1	0.067%
Severe tricuspid valve damage (by 2 or 3 degrees to grade 4)	11	0.733%
Double (hemopericardium rescue cardiac surgery, tricuspidvalve damage)	1	0.067%
All patients	1500	100.0%
**Types of cardiovascular damage in 21 patients**		
RAA wall tear (one double)	8	0.53%
Tear of connection of RA to SVC	5	0.33%
Tear of VCS (in 2 symptoms occurred after lead removal and when guide wire or new lead passed to right pleura)	3	0.20%
Rupture of connection of RAA to RV (partial RAA rupture)	1	0.067%
Tear of lateral wall (double)	1	0.067%
Tear of RA and CS	1	0.067%
Tear of connection of RA to IVC	1	0.067%
Tear of RV	1	0.067%
All patients requiring surgical intervention (one pericardiocentesis only)	21	100.0%

Abbreviations: BP—blood pressure, CS—coronary sinus, IVC—inferior vena cava, RA—right atrium, RAA—right atrial appendage, RV—right ventricle, SVC—superior venacava.

**Table 5:** Description of the symptoms of cardiac and vascular damage during TLE.

Clinical Course and Hemodynamic Changes since Symptom Onset to Bleeding Cessation	Number of Patients	Max Drop in Blood Pressure [mmHg]	Max HR Change [per minute]	Max Decrease in CO_2_ [mmHg]	Global Loss of Blood Volume [mL]
RAA wall tear (one double)	8	52.82 ± 25.13	6.15 ± 13.81	3.82 ± 2.82	1628.8 ± 2164.8
Rupture of connection of RAA to RV (partial RAA rupture)	1	30	5	5	800
Tear of connection of RA to SVC	5	53.73 ± 25.92	18.32 ± 7.62	7.01 ± 2.03	2200.0 ± 1881.4
Tear of lateral wall: double	1	70	−20	1	1500
Tear of RA and CS wall	1	55	10	5	5000
Tear of connection of RA to IVC	1	73	0	3	600
Tear of SVC wall (in 2 cases after lead removal and when a guide wire or new lead passed to the right pleura)	3	65.33 ± 27.23	10.32 ± 3.37	4.12 ± 3.27	3200.4 ± 1453.2
Tear of RV wall	1	75	0	6	1100

Abbreviations: BP—blood pressure, CS—coronary sinus, HR—heart rate, IVC—inferior vena cava, RA—right atrium, RAA—right atrial appendage, RV—right ventricle, SVC—superior vena cava.

**Table 6:** Analysis of tricuspid valve function before and after TLE.

**Tricuspid Regurgitation Before TLE**		
Degree of tricuspid regurgitation	No. of patients	%
Lack (grade 0)	58	3.91%
Mild (grade 1)	734	49.43%
Moderate (grade 2)	323	21.75%
Intermediate (grade 3)	256	17.24%
Severe (grade 4)	114	7.68%
All	1485	100.0%
Lack of examination, not described	15	
Mild (0,1)	792	53.33%
Moderate/intermediate (2,3)	579	38.99%
Severe (4)	114	7.68%
All	1485	100.0%
Lack of examination, not described	15	
**Changes in TR after TLE**		
Direction of changes in TR	No. of patients	%
No changes	1175	79.61%
Increase by 1 degree	106	7.18%
Increase by 2 degrees	35	2.37%
Increase by 3 degrees	8	0.542%
Decrease by 1 degree	131	8.87%
Decrease by 2 degrees	21	1.42%
All examined patients	1476	100.0%
Lack of examination, not described	24	
**Worsening tricuspid regurgitation after TLE**		
	No. of patients	%
Rupture of chordae tendineae	47	3.18%
Moderate increase in TR by 2 or 3 degree but not to grade 4	31	2.10%
Significant (by 2 degrees) increase in TR to grade 4	12	0.813%
**Management of TV damage as TLE major complication**	**No. of patients**	**% among 12** **(% among 1500)**
TV replacement (2 acute, 3 late)	5	41.67% (0.33%)
Classified as refused operation but general condition unchanged	3	25.00% (0.20%)
Not classified, remained under observation	3	25.00% (0.20%)
Disqualification—cancer disease	1	8.33% (0.067%)
All	12	100.0%

Abbreviations: TLE—transvenous lead extraction, TR—tricuspid regurgitation, TV—tricuspid valve.

**Table 7:** Classification of tricuspid regurgitation after TLE.

Worsening TR after TLE	Number of Patients	TR before TLE	TR after TLE	% (*n* = 149)	% (*n* = 1476)	Present Classification	Suggested Classification
Increase by 1 degree	27	0	1	18.12%	1.83%	Lack	Lack
	38	1	2	25.50%	2.57%	Lack	Lack
	25	2	3	16.78%	1.69%	Lack	Minor
	16	3	4	10.74%	1.08%	Lack	Minor
Increase by 2 degrees	1	0	2	0.671%	0.067%	Minor	Minor
	30	1	3	20.13%	2.10%	Minor	Minor
	4	2	4	2.68%	0.27%	Major	Major
Increase by 3 degrees	8	1	4	5.37%	0.54%	Major	Major
All (*n*, %)	149			100.0%			

Lack—lack of complication, Minor—minor complication, Major—major complication.

## Data Availability

Readers can access the data supporting the conclusions of the study at www.usuwanieelektrod.pl (accessed on 11 September 2021).
